# Policy-makers’ views on translating burden of disease estimates in health policies: bridging the gap through data visualization

**DOI:** 10.1186/s13690-021-00537-z

**Published:** 2021-02-04

**Authors:** Amelia Lundkvist, Ziad El-Khatib, Nikhila Kalra, Tomas Pantoja, Katherine Leach-Kemon, Christian Gapp, Tanja Kuchenmüller

**Affiliations:** 1 Department of Learning, Informatics, Management and Ethics (LIME), Karolinska Institutet, Stockholm, Sweden; 2 Department of Global Public Health, Karolinska Institutet, Stockholm, Sweden; 3 World Health Programme, Université du Québec en Abitibi-Témiscamingue (UQAT), Québec, Canada; 4 Institute for Health Metrics and Evaluation (IHME), University of Washington, Seattle, WA USA; 5 Pontificia Universidad Católica de Chile, Santiago, Chile; 6 World Health Organization Regional Office for Europe, Copenhagen, Denmark

**Keywords:** Knowledge translation, Data visualization, Policy officers, Burden of disease

## Abstract

**Background:**

Knowledge Translation (KT) and data visualization play a vital role in the dissemination of data and information to improve healthcare systems. A better understanding of KT and its utility requires examining its processes, and how these interact with available data tools and platforms and various users.

In this context, the aim of this paper is to understand how relevant users interact with data visualization tools, in particular Global Burden of Disease (GBD) visualizations, while also examining KT processes related to data visualization.

**Methods:**

A qualitative case-study consisting of semi-structured interviews with eight policy officers. Interviewees were selected by the Institute for Health Metrics and Evaluation (IHME) from three countries: Canada, Kenya and New Zealand. Data were analyzed through framework coding, using qualitative analysis software.

**Results:**

Policy officers’ responses indicated that data can prompt action by engaging users, and effective delivery and translation of data was enhanced by data visualization tools. GBD was considered valuable for use in policy (e.g., planning and prioritizing health policy; facilitating accountability; and tracking and monitoring progress and trends over time and between countries). Using GBD and data visualization tools, participants quickly and easily accessed key information and turned it into actionable messages; engaging visuals captured decision-makers’ attention while providing information in a digestible, time-saving manner. However, participants emphasized an overall disconnect between research and public health. Functionality and technical issues, e.g., absence of tool guides and tool complexity, as well as lacking buy-in and awareness of certain tools from those less familiar with GBD, were named as major barriers. In order to address this “know-do” gap, user-friendly knowledge translation tools were stated as crucial, as was the importance of collaboration and leveraging different insights from data generators and users to inform health policy.

**Conclusions:**

Policy officers aware of KT are willing to utilize data visualization tools as they value them as an engaging and important mechanism to foster the use of GBD data in policy-making. To further facilitate policy officers’ efforts to effectively use GBD data in policy and practice, further research is required into how users perceive and interact with data visualization and other KT tools.

**Supplementary Information:**

The online version contains supplementary material available at 10.1186/s13690-021-00537-z.

## Background

Timely, accurate and high-quality health information is one of the key inputs for public health action, health systems strengthening and progress towards the achievement of important health goals, such as universal health coverage and the health-related Sustainable Development Goals [1]. Effective public health action and implementation of useful health policy is integral to achieving improved health. A range of tools and mechanisms are used to promote the utilization of health information in policy development and implementation. They can be summarized under the name of knowledge translation (KT) approaches [2].

The World Health Organization (WHO) defines KT as: “the exchange, synthesis, and effective communication of reliable and relevant research results” [3]. The focus of KT is on promoting interaction among the producers and users of research, removing the barriers to research use, and tailoring information to different target audiences so that effective interventions are used more widely [4]. WHO identifies four key KT approaches that, either singularly or combined, illustrate the link between health information and action [5, 6]:

- Push approaches, where information producers ensure research processes and findings are more digestible for decision-makers by effectively translating technical information and jargon into non-technical information;
- Pull approaches, where information users, such as policymakers, request health information based on their needs;
- Exchange approaches emphasizing linkages between information producers and users, which can be facilitated by knowledge brokers (KBs), to work together at specific points or during the entire information generation cycle; and finally,
- Integrated approaches, where a KT platform is established in an organization or broader health system allowing the promotion of early and sustained engagements between information producers and users and institutionally linking research to action through push, pull or exchange efforts [6].

These approaches have been used by information producers, policymakers and others in the health sector in countries across the world, engaging many different types of stakeholders in diverse areas of policy-making [5].

In the context of KT, health information includes the summary and analysis of health status and problems in populations over time, as well as quantification of associations between outcomes and risks; it also assesses effectiveness of public health interventions [5]. Data consist of raw facts of a study or work, in qualitative or quantitative form; while evidence is defined as ‘findings from research and other knowledge that serves as useful basis for decision-making in public health and health care’ [5]. Explicit knowledge consists of these three: health information, data and evidence [5]. Health data are usually presented in different formats and with different degrees of aggregation, which can make understanding it challenging. This was one of the issues that has been identified as a barrier to participants’ use in decision-making processes.

Data visualization—understood as ‘the representation and presentation of data to facilitate understanding’ – is considered an important mechanism for increasing the usefulness of health information [7, 8]. Data visualization can be considered a ‘push’ approach to KT [9] as it seeks to present data in more accessible ways, such as graphs, charts, and maps with interactive features [10], but it also facilitates interaction between multisectoral stakeholders from different areas by connecting them in the production of such tools, resulting in mutually beneficial collaboration [11]. Given this, data visualization tools can play an important role in KT processes.

The usefulness of data visualizations has been investigated and critically assessed in computer science for some time [12], but so far there is limited understanding of how well existing visualization tools help users to master the wealth of information represented by complex data. This is particularly relevant for health sector and health policy systems decision-making. Providing a base for further assessments, this paper explores how a number of professional experts in a few countries analyze and use Global Burden of Disease (GBD) data using data visualization platforms.

This study aims to contribute to a more robust understanding of the use of data visualization to support KT processes. It uses a case-study of policy officers’ engagement with one data visualization platform, GBD Compare. GBD Compare is a tool developed as part of the GBD study, led by the Institute for Health Metrics and Evaluation (IHME) in collaboration with thousands of researchers worldwide. GBD quantifies global health loss from diseases, injuries, and risk factors across age groups, sexes, countries, regions, and time, with estimates currently produced for 195 countries, by age and sex, from 1990 to the present. GBD Compare is primarily a push approach aimed at making research processes and findings more digestible for decision-makers.

The GBD study provides a rich context for exploring processes of knowledge translation, due both to the vast scope of the data available and its explicit orientation towards helping policymakers understand their countries’ health challenges. In order to support decision-making, GBD Compare has been developed as a KT mechanism for this audience, with the aim of “allow [ing] decision-makers to compare the effects of different diseases, such as malaria versus cancer, and then use that information at home” [13]. The GBD estimates are intended to be used for policy and so KT is an integral part of this effort; if people are not able to access, interact with, and understand the estimates, it is difficult to incorporate them into decision-making. GBD Compare is intended to help users explore patterns and trends, make comparisons across and between different axes such as country, demographic groups, and diseases, and create and capture relevant graphics such as maps, graphs, and tables. Given its range and intended orientation towards supporting policymaking, GBD Compare provides a useful case study for exploring the following questions:

1. How do policy officers perceive, interpret and utilize web-based data visualization tools?
2. How can these tools and platforms be optimized as catalysts for KT processes?

## Methods

### Study design, setting and methods

This was a qualitative case-study using semi-structured interviews with policy officers (see Additional file 1 for the interview guide). A case-study design was selected due to the descriptive and exploratory nature of the inquiry—how knowledge translation tools are perceived, interpreted, and used by policy officers—and because understanding the specific context in which these processes are occurring is important to answering the primary research questions [14]. Given this study design, rather than seeking to draw general inference about larger populations or other contexts, this work seeks instead to examine and explicate processes related to the use of data visualization for knowledge translation, which may have relevance, explanatory value, or provide transferrable lessons for other settings.

Semi-structured interviews allowed data collection around themes pertinent to the research topic while also giving respondents the space to discuss issues or areas that they deemed important [15]. The purposive selection was facilitated by IHME using a predetermined list of potential respondents with expertise and characteristics relevant to the study (policy officers who advise on public policy in government settings in a technical capacity; individuals who have prior familiarity with the GBD and its data visualization platform, GBD Compare; and English-speaking). The final sample size was determined by reaching saturation [16].

### Study sample

As described above, the study sample consisted of respondents who were a) policy officers and b) familiar with the GBD tool. In terms of their involvement in policymaking, we define policy officers as people who influence policy processes in order to improve and shape implementation strategies. These advisors often act as KBs between the academic world and policymakers by providing data and evidence to governments. Working as developers and promoters of new government policy, these specialists have technical expertise in a specific field and advise colleagues (e.g., senior bureaucrats and government ministers) on policy options. Among other functions, they help evaluate and monitor policies, create dialogues, and strategize.

A total of eight persons, out of 10 invitees, agreed to participate in a semi-structured interview. Seven interviews were conducted over a period of 3 months (with one done as a joint interview with two participants). Of the seven interviews, five were Skype audio, one Skype video, and one Zoom video call. Interviews ranged from 25 min to 64 min long, although a majority were about 50 min in duration. The study participants included an epidemiologist, a research scientist and research associate, a UHC program coordinator, a Monitoring and Evaluation specialist, an NCD Division Lead, a principal advisor in epidemiology and principal policy analyst in strategy and policy. The average interview duration was 50 min. Seven interviewees were female and one male with a wide range of duration of work experience in the field of health policy and/or knowledge translation work (range 3 to 15 years). All participants were highly educated individuals holding senior positions, having either master’s degrees (*n*=7), or PhD (*n*=1), in public health and medical sciences. Three currently work in healthy policy and were previously clinicians before transitioning into policy-focused roles.

To explore potential contextual dynamics, respondents with these characteristics were also selected from different countries with varied data landscapes and policy contexts. In this case, one Low- and Middle-Income Country (LMIC) (Kenya) and two high-income countries (HICs) (Canada and New Zealand). Each participant was an individual with expertise in a particular discipline and employed by a government agency working in different ways to contribute to implementing health policy. The interviewer was located in Stockholm, Sweden, while interviewees were located in their respective countries’ locations.

### Data management and framework analysis

All interviews were recorded after receiving participants’ consent. Interviews were then transcribed and uploaded to qualitative analysis platform, Dedoose, for data analysis [17]. Data analysis began using open coding as a process to ‘open up’ the text in order to discern ideas and meanings [18]. This consisted of close readings of interview transcripts and consideration of the multiple meanings found within them [19], generating and applying codes, and then looking across codes to discern key themes [20]. These themes were then grouped by adapting Lavis et al.’s framework for assessing country-level efforts to link research to action [6]**.** Their framework consists of four elements: general climate for research use, production of relevant and synthesized research, mix of activities linking research to action and the evaluation of efforts to link research to action [6]. This framework explicates the various directions, dynamics and inter-relationships of those involved in KT and data use. Of note, while Lavis et al’s framework was used, another theme, data use, was added to encapsulate all results.

## Results

### Themes

**Fig. 1:**
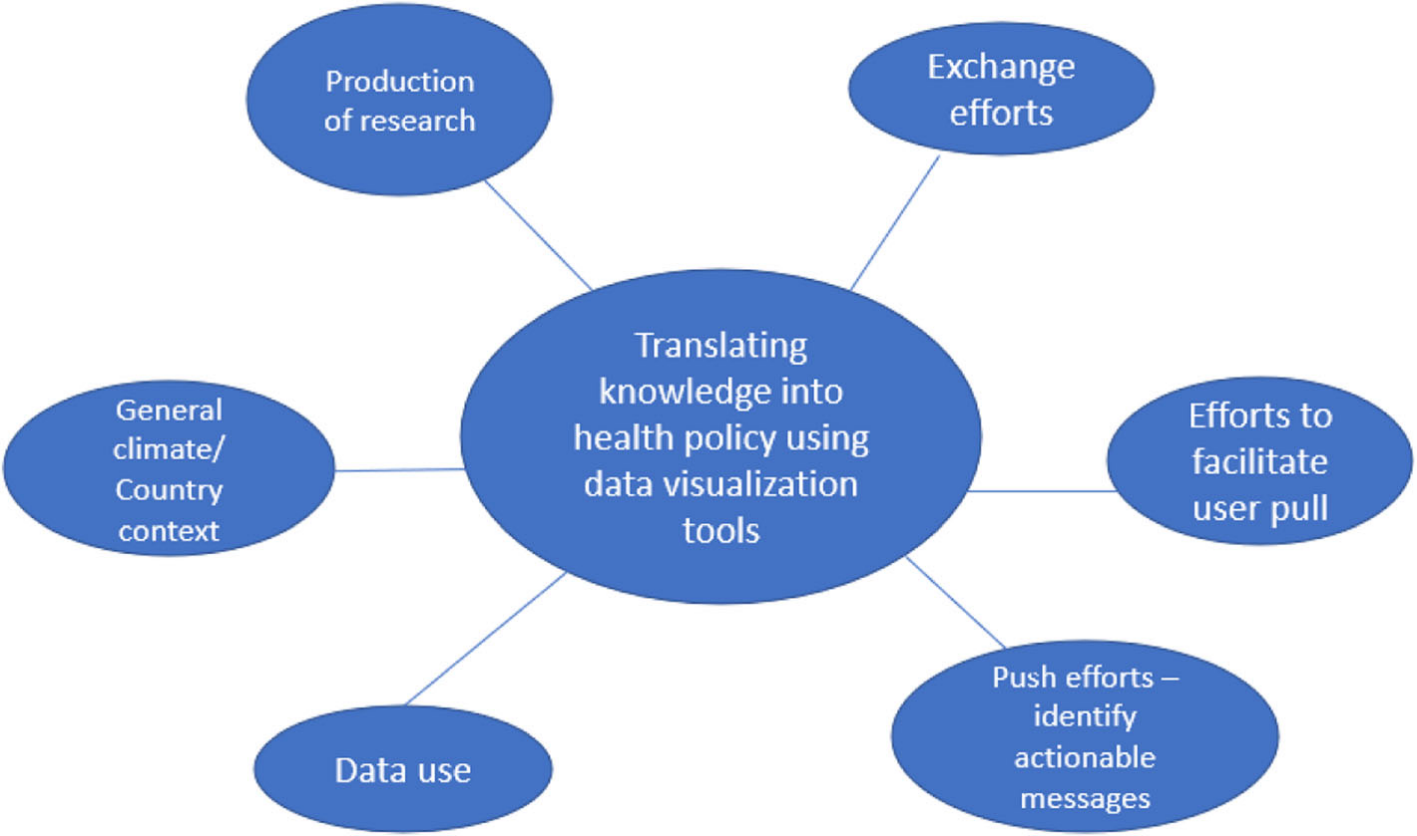
Themes

A total of 6 themes and 16 subthemes emerged from the data (see Table 1). Participants identified different advantages and weaknesses of data visualization tools for KT, as well as discussing various aspects of knowledge translation and data visualization use. Specifically, the primary themes were: production of research, exchange efforts, efforts to facilitate user pull, push efforts, general climate, and data use (see Fig. 1):

**Table 1:** Components of the data visualization

Text of Themes (*n*=6)	Subthemes (*n*=16)	Quotes from participants
Exchange efforts – build relationships among researchers and research users who have shared interests	1. Multisectoral collaborations and consolidated efforts enhance information exchange	Need for multisectoral collaboration and communication between data generators and users to produce data, foster data use and inform health policy; more interlinkages and exchange needed between GBD collaborators, which include policy-makers, analysts and other data users; researchers need to explain data effectively so wider audience can understand in order to facilitate exchange, otherwise meaning of information will be lost; bringing people together e.g. conferences or trainings valued as helpful and important; gaps seen between academia and public health realms, which can be bridged by collaboration; collaborations between departments, such as Ministry of Health and Ministry of Education seen as crucial to make best policy; barriers include lacking time and resources to facilitate these partnerships
Efforts to facilitate user pull – relevant information optimally packaged	2. Interactive nature of data visualization tools facilitates data use, accessibility and understandability	Data visualization tools engage users and their audiences with interactive nature and ease of use, making data more useful; eye-catching aspect of visualizations makes impact by grasping users’ attention
	3. The GBD Compare tool offers a variety of functions meeting the user’ needs	GBD tools facilitate the comparison between countries and counties over time and DALYs; GBD tools provide subnational level data and show risk factors; GBD identifies gaps in data and can help to target areas that need further attention; use data visualization tools for quick access to and delivery of data
	4. Complexity of using GBD Compare can inhibit data access and use	GBD Compare requires regular use to understand and maximize utilization; users report more guidance and training needed to use GBD Compare as insufficient user understanding of GBD Compare is limiting
	5. Missing functionalities and technical difficulties of GBD visualization tools limit usability	Lacking ability to look at all causes of disease and customization functions in tools, such as saving previous searches; GBD tools lacking subnational level data for certain countries; difficulty downloading SDG charts; need to repeatedly run searches as may not work first time; GBD tools not compatible with all browsers; GBD tools need to be adjusted and updated
	6. User engagement enhanced with GBD oversight team	GBD oversight team of deputy directors-general provides technical and/or policy assistance to both policymakers and researchers on GBD Compare, which aids in maximization of tool use; importance of GBD advisory entities highlighted by users
	7. GBD methodology education and training yields more buy-in and engagement	Need to increase GBD engagement at local level in settings where users lack buy-in, knowledge and/or methodology as increased buy-in once benefits of GBD seen
	8. Increased guidance and recommendations needed from IHME on how to use GBD data in policy	More guidance requested as lacking explanation for global patterns and recommendations for potential policies that could be implemented, such as examples of successful policy interventions for certain diseases e.g. meta-analysis
Push efforts – identify actionable messages	9. Data visualizations utilized as means to communicate data	Use data visualization to better understand data by quickly, uniquely and clearly conveying information; visualizations prompt action and support policy recommendations to stakeholders by grabbing their attention and illustrating data
	10. Data visualization tools act as valuable complementary source in coordination with local tools and/or systems	Data visualization tools identify and fill gaps in local information or data; GBD tools used with other methods, such as coding, when local data not available
Production of research – priority-setting processes	11. Transparency and consistency of data sources needs to be maintained	Users highly value and appreciate transparency of data sources as data is more trustworthy; discrepancies between GBD and local data and within GBD data cause confusion and distrust among users so less willing to use data
General climate – support and value placed on linking research to action	12. Adequate awareness, resources and time should be devoted to KT efforts as KT acts as catalyst for production of evidence that informs and supports policy recommendations	Insufficient time, awareness and/or resources contribute to less effective KT; KT should be prioritized among many factors and competing needs involved in decision-making as more likely to result in action; funding contingent on quality of researchers’ KT plan incentivizes prioritization of KT; KT valued as important process leading to informed decisions and make stronger policy recommendations; KT important in facilitation of evidence accessibility and understandability; throughout data generation, consider KT to provide better information
	13. Overall lacking buy-in and awareness of GBD and data visualization tools from country leadership can lead to minimal utilization	GBD and/or data visualization not known, incorporated or used by policy-makers in some settings as certain country leaders resistant to using outside data and skeptical of GBD estimates, particularly in lower income countries; leadership attitudes or unawareness negatively affects user interest
Data use	14. GBD data used to forecast, plan and prioritize health policy	GBD data used to predict future needs and prioritize resources accordingly; GBD data perceived as important part of informing decisions, policymakers and public and ultimately eliciting change; data used to monitor and track progress over time and justify resource allocation and funding
	15. Facilitate and improve accountability; track and monitor progress and trends over time and between countries	Comparison between countries illuminates gaps and thus triggers action; GBD results inform and discern patterns within and among countries

### Exchange efforts – build relationships among researchers and research users who have shared interests

Participants from both LMIC and HICs highlighted the need to collaborate and consolidate efforts between data generators and users to produce data, foster data use and inform health policy. Participants emphasized the importance of collaboration in utilizing and incorporating different insights, perspectives, and experiences. They described the interlinkages and exchange between all involved parties as crucial in informing health policy: participants highly valued conferences, trainings or other events that brought people together so they could meet, discuss and exchange ideas and information with one another. Given the explicit intent of GBD data to be used as a decision-making tool and to inform health policy, respondents noted the imperative of a collaborative approaches to using tools, engaging in dialogue with colleagues rather than simply extracting and presenting data to them. As one respondent, a policy analyst in a high-income setting, explained about using GBD Compare with a colleague: “Both of us come from different perspectives … so I’m mainly using the visualization tools and also using the methodology behind it to create epidemiological insight … and then provide it and discuss it with [colleague], so that he can look at it from the policy perspective to see what has been done in the field before.” Other respondents noted the importance of constant communication between different loci of decision-making when engaging with health data like that from the GBD, which has implications for many sectors; for example, a participant from an LMIC highlighted the need to make connections between different areas, particularly between and amongst various Ministries, such as Transport and Finance, when thinking about audiences for the information provided in the tools. Similarly, a research scientist and an epidemiologist from HIC stressed how delivery of data needs to be done thoughtfully and, importantly, to the right people, with one explaining: “you can’t just show them the evidence like here’s the reality … it’s really a process of change and so it’s figuring out who your stakeholder, where your stakeholders or your decisionmakers are right now”. Applying the tools therefore helps in the process of fostering data-driven decision-making, but only if relevant entities and actors are identified and brought in.

With reference to collaboration, participants noted a key challenge that remains in terms of a broader disconnect between research and public health. Respondents noted that without effective communication and collaboration between relevant stakeholders, health information can remain under or unused. An epidemiologist, from LMIC, put it succinctly: “people don’t talk to each other, academicians and then the ward and the public health professionals and so there is a gap there that’s a kind of, the bridge, the connection is not there”. While the GBD study seeks to use a collaborative process in the production of the data that ends up in GBD Compare, this finding suggests that participants remain acutely aware of the underlying challenges of collaboration and cross-stakeholder communication remain when it comes facilitating effective KT processes.

### Facilitating user pull – technical considerations

Participants from LMIC countries noted certain challenges in using the tool, GBD Compare. In particular they noted the complexity and intricacies of the tool, its scope and interface requiring regular use in order to understand and maximize utilization, and the way data is presented in graphics sometimes proving difficult to interpret. As one participant noted, “if I’m seated at my desk and I have no training and I want this information, I may not be able to make sense of that information”. A research scientist from HIC observed some did not utilize the tools if they did not feel as confident using them or as familiar with how to use them. More guidance and training were highlighted as necessary for increasing utilization of the tool, as without clear guidance, insufficient user understanding of GBD Compare can be quite limiting. In addition, participants from all three countries suggested several improvements that could be made to tool functionality, ranging from technical issues, such as ensuring compatibility with certain browsers, to the way in which information is displayed.

### Facilitating user pull – research production

While respondents noted challenges in using the data visualizations, they all identified deeper structural issues at the local and country-level hindering GBD uptake by various policy officers and country leaders, such as lacking buy-in, or awareness, of GBD. Those from LMIC settings observed a need to increase engagement around GBD among policy and other audiences in country, suggesting that buy-in would increase once the benefits of GBD could be seen and the methodology was better understood. Participants experienced challenges garnering interest in using the tools, and commented on a certain skepticism and questioning of GBD data and its quality, particularly in the context of existing and competing information sources. One participant from LMIC explained that “the country has invested a lot in our routine data system that anytime you could bring this issue and not and mention that the routine data has not been used, it could immediately face a lot of resistance.” Another noted, “sometimes people feel that evidence that is not generated in-country is not good enough.” The same concerns arose from those in high-income settings. For instance, skepticism surrounding the estimates and how they were calculated raised some doubts. A research scientist in HIC noted these concerns: “one barrier we have with using it internally is sometimes our more senior managers are concerned that because these are modeled estimates, they’re not going to match what comes out of our national systems”.

Moreover, while participants highly valued and appreciated transparency of data sources in the GBD Compare tool and found that they themselves and their colleagues were more trusting of data if there was clarity about its source, several users discussed concerns about discrepancies found within GBD data, which is re-estimated each cycle due to new methods and underlying data sources: “the little issue that at times we have is the fact that each and every time every year when the new GBD results are released, you may find varying … indicators”. Confusion regarding modeling methodology and estimates led to distrust among users and were therefore less willing and likely to use the data. Despite these concerns, participants had hope that visualizations could help gain more trust once GBD methods were shared and explained.

In one HIC, the presence of a GBD oversight team was found to be of immense help. The oversight team consisted of deputy directors-general who provide technical and/or policy assistance to both policymakers and researchers on GBD Compare. This led to more, maximized tool use, and its value was emphasized highly by participants who worked with this team. However, more guidance from IHME was requested around explanations for global patterns and recommendations for potential policies that could be implemented. Participants hoped that examples of existing, successful policy interventions for certain diseases, e.g., increasing obesity rates, could be shared by IHME.

### Push efforts – identify actionable messages

Data visualization was used to better understand data by quickly, uniquely and clearly conveying information. Visualizations were noted as a valuable method to prompt action and support policy recommendations to stakeholders by grabbing their attention and providing illustrative data. As one policy analyst from a HIC shared*:* “decisions are not rational things, are they? They’re about hearts and about minds and so visualizations are something that helps you to bridge the heart and the mind a little bit … images can stay with you in a way that words on a page won’t, so I think there’s something about the translation that’s hard to encapsulate but it helps to tell the story.”

Policy officers indicated that data can prompt action by engaging users, if compiled and presented in a succinct, colorful way with the help of data visualizations and related tools. Instead of handing users a bland 60-page report, policy officers could present the data in a more productive manner. One respondent from an HIC described the power of data visualizations, in particular the GBD tree map visualization: “that one is really useful for, you know, showing the overall burden and then making a comparison to resources allocated. Whenever I present that slide, I get silence in the room for 30 seconds and then discussion”.

Policy officers also noted that being able to present data on risks factors and their contribution to disease was useful for capturing audience attention, challenging preconceptions of the current situation and therefore spurring appropriate, effective action. Making the evidence more engaging was perceived as critical to incite and inspire change in policymaking, with one HIC policy analyst noting that “it’s quite a powerful way of describing the interplay of risk factors and poor outcomes for health, and just yesterday we were using this analysis for some of our senior leaders in our section to look at health loss and the factors that contribute to it. So, I’d say the visualization tools are very important part of bringing the data to life.”

Additionally, user-friendliness emerged as an important aspect of data visualization and its powerful, unique ability to convey information. Participants from LMIC discussed “scorecard” approaches to presenting information on GBD Compare as attention-drawing tools: “it’s color coded so you have like red, orange and green depending on the progress that has been made … it can be printed, but most of the time, you can even get it on your mobile phone”. In the words of another participant, at the click of a button you can have information and indicators for an entire country. Top management, or other audiences who need to know the important facts, do not need to spend a lot of time trying to access this kind of data: “they just need it at a glance, they are so busy”.

While, as discussed earlier, existing information systems could be seen as a barrier to buy-in for the data found in GBD Compare, it is also the case that participants have been able to show the utility of GBD and its visualization tools as a useful complementary resource in conjunction with existing local systems, and using the tools to identify and fill gaps in local information or data proved very valuable. For instance, a research scientist used GBD data in addition to their existing platforms. They do not have a system that comprehensively covers both morbidity and mortality for all causes. In LMIC, a participant described using GBD for subnational estimates: “for example life expectancy, or top five causes for mortality … we could easily sell that even to the leaders at the subnational level”. The GBD estimates are therefore used as a way to compare in-country data to give a better overall grasp of the situation, used in tandem with other systems.

### General climate – country support and approach (es) towards KT

According to participants, in order to optimize KT, sufficient resources, awareness and time all need to be devoted to the process of KT and policymaking, and a lack of awareness and engagement with GBD and/or data visualization were identified as issues to be addressed addressed. These issues were particularly seen in the LMIC setting, where country leaders were resistant to using outside data and more skeptical of GBD estimates. Leadership attitudes set the tone for the rest of the country and trickled down into departments or divisions. In general, respondents noted that fewer resources were devoted to KT and data collection and visualization in LMIC countries, highlight the need for “more money, more funds, more capacity … especially in my field …. there’s a lot of gaps in knowledge”. In high-income contexts, however, KT was much more supported. For instance, using KT was even enforced as a part of the research and funding process in these contexts. A research scientist in HIC explained how KT is an integral part of acquiring funding and how a KT plan is mandatory and evaluated as part of the funding application process.

### Data use

GBD data was used to help identify needs and prioritize resources accordingly. Participants described how GBD helps inform the future, for instance by giving direction to the country’s goals. One respondents explained that “it shows you how [things have] changed and you can change over the years, to see how both the risk and the causes of diseases are changing … you can see where you’re making progress and where we are not making progress and how, if that’s a neglected area, we should focus on it.” Policy officers found that GBD results, as presented in GBD Compare, help to discern patterns, and could therefore be used for not only monitoring, but also planning for time, funding and resource allocation:

“We have the indicators which we use to monitor the progress of the project, so each and every time we have to keep looking at the data, see how the data is behaving, look at trends over time, maybe over the months, over the years, and see whether, make decisions based on that and see if we are on track you know if we need to do, need to change our tactic for better results.”

A research scientist in HIC further explained commented on their utility for planning, explaining how in “planning, developing our five-year plans, we have to actually look back and really bringing all that still, the charts and you know, the visualizations to show the current situation—it becomes like building your case,” though also emphasized that other aspects of decision-making need to be considered, as “there are many factors that influence what an organization is going to focus on and evidence is just one thing.”

Another key aspect of GBD Compare highlighted by respondents was how the data facilitated comparison among countries, which illuminated any gaps and may trigger processes of policy changes. While GBD data in itself does not indicate what the policy responses should or would be, it helps to identify gaps, understand areas where disease burden is high or significantly increasing, particularly in this directly comparative context.

Data was also used to increase accountability and transparency. In this case, participants noted one other tool developed by IHME, Financing Global Health, a data visualization focused on tracking and monitoring government spending on health. One respondent noted that this tool was helpful in conjunction with GBD Compare:

“You can say the US government spends this much on malaria … I think that’s really good because many times even for us, being right now we are lower middle-income country but when we were low-income country, there was a lot of investment by partners, you know donors … you could have this notion that they spend this amount but that amount doesn’t really get to the ground.”

## Discussion

Our findings indicate that while there were variations in the perception, interpretation and utilization of a data visualization tools by policy officers, all study participants found these tools to be extremely useful.

Data visualizations are instrumental to quickly and clearly conveying information and thus contribute to increasing the use of burden of disease data in policy. According to our findings, the process of KT requires sufficient resources, awareness, time and sense of ownership. In some cases, participants perceived unwillingness to use GBD data among their colleagues and the wider stakeholder community. Knowledge producers, such as IHME, can make more concerted efforts to increase buy-in and familiarity with various data platforms, perhaps by advertising their objectives and their data more in the health information realm, and by providing more training for current and prospective users.

A substantial body of literature confirms that KT processes build important bridges between evidence and practice, inter alia through co-creation process and the involvement of all relevant actors, from differing backgrounds [21]. These results build on existing evidence of the literature on data visualization and KT, such as assertions that data visualization’s interactive nature allows decision-makers to explore data from various perspectives [22]. Scholars have highlighted how visualizations can help users (e.g. policy officers presenting data and information) explore, analyze and communicate results from complex big data in health [22], and can be used to convey a larger story or narrative in a stimulating and more effective way than text alone [23].

However, there is a scarcity of research on using information technology as a means for knowledge dissemination in health policy-making [24]. A systematic review by Delnord et al. reports that only one previous study proposed a framework for monitoring the impact of national health information systems in regards to KT [25]. During the study process, we observed that inaccessibility of the evidence hampered evidence-informed policy; evidence will be quickly discarded by policy-makers if they struggle to access it online or if the way in which is presented is difficult to understand [26]. However, data visualizations tools represent just one example of strategies for facilitating the use of explicit knowledge (health information, data, evidence) in policy. Others include synthesis tools, such as policy briefs, which summarize and present information in a more accessible ways, as well as knowledge networks (e.g., regular meetings or advisory groups/committees) that bring stakeholders together to exchange ideas [9].

Actors’ use of available interactive tools and the creation of exchanges and exchange between data producers and users affect the extent to which health information is applied in the policy process [27]. Collaboration between multisectoral stakeholders such as evidence producers and users, funders and actors in healthcare with varying perspectives increases mutual understanding and trust which facilitates KT [26, 28], which is echoed by participants’ emphasis on the importance of collaboration and communication.

The three countries in this study provided contrasting contexts in regards to both GBD uptake and health information systems, in terms of resources, data quality, transparency as well as awareness and support for KT. This is particularly relevant for public health in LMICs, where local-level evidence might be ignored or inadequate hardware and data quality prevail [29]. In Kenya, a majority of health facilities are still operated by faith-based organizations and non-governmental organizations, data remains scarce and KT is not prioritized [30]. In contrast, policy-makers in HICs have a wealth of data, resources and they integrate KT and GBD data in their policy-making process [21, 22]. While some participants noted attributing high importance to KT and use of data visualization, they also encountered hindrances such as lacking resources, particularly in LMICs. Dedicated resources (e.g. staff, time and money) to support KT efforts are needed [31].

In order to take appropriate actions, health policy-makers require various kinds of information about health system performance and public health problems and needs [24]. Health information and data helps to identify problems, as well as aids monitoring and evaluation by measuring the magnitude of a disease and assessing progress in addressing these health problems [5]. However, data is only part of the evidence that is needed for effective evidence-informed policy-making. For the remainder of the policy cycle, other kinds of evidence and health information are needed, in particular primary and secondary research [5]. The Evidence-informed Policy Network (EVIPNet), of WHO, is looking at comprehensively addressing the evidence needs of policy-makers at all stages of the policy cycle while synthesizing the best available evidence in a user-friendly manner [5].

### Methodological considerations

This was a qualitative case study that generated an in-depth, multi-faceted understanding of data visualization in real-life contexts across three countries. The case study provides valuable input from selected experts to help design further research; specifically, the usability and relevance of sophisticated visualization tools in the analysis of complex data and information like GBD. However, such an approach comes with its own methodological limitations, including selecting study participants purposively (e.g., pre-selected by IHME based on participant use of GBD Compare); also, findings may not be generalized to other contexts [32].

The research questions were exploratory in nature, seeking specifically to understand and draw out inferences from respondent perspectives and experiences. An inductive approach in this case is optimal, where data was gathered to build concepts, hypotheses, and theories, rather than deductively deriving hypotheses to be tested [33]. Semi-structured interviews allowed for participants to diverge and proved useful as relevant topics could be delved into and discussed in detail. Also, participants came from different backgrounds, professions, and countries. Interviewing policy officers from three countries with contrasting contexts regarding GBD uptake, and healthcare systems generally, yielded diverse responses and data while also giving the study a richer, more comparative perspective.

Of note, interviews and data collection were not done in situ in each country where participants were doing their work. A better grasp of the local system and context would have provided more background and better understanding of the participants’ responses and emerging themes from their insights. Additional limitations include self-reporting and potential biases, the main bias being the low number of interviews, as well as the fact that interviews may take different courses, so it is difficult to compare responses.

## Conclusions

This study provides insight into the complexities of utilization and interpretation of data visualization tools. For instance, policy officers familiar with the concept of KT are more willing to utilize data visualization tools as they view them as engaging and important implements for KT and policy-making process. While future research can build on these observations to examine how users perceive and interact with data visualization and related tools, and their implications for KT, a suggestion emerging from this work is for organizations such as IHME and WHO to consider how certain KT mechanisms can be strengthened and thus make data more digestible, usable, and informative for health policy-making. Focus should be placed on finding and developing solutions that facilitate efforts in bridging the gap between evidence and policy; specifically, understanding how health metrics are used and interpreted and developing a KT framework incorporating these can help identify and overcome challenges. The results of this study also demonstrate the importance of actors across the health information system coming together to work with one another in processes of knowledge translation. Thus, adopting a system-wide approach when translating health information into policy could be useful to strengthen and enhance a collaborative research processes and encourage systematic, transparent use of GBD in the policy-making process.

## Supplementary Information

**Additional file 1.**

## Data Availability

Additional data can be obtained upon request.

## Abbreviations

EVIPNet: Evidence-informed Policy Network
GBD: Global Burden of Disease
HIC: High-Income Countries
IHME: Institute for Health Metrics and Evaluation
KB: Knowledge Broker
KT: Knowledge Translation
LMIC: Low- and Middle-Income Countries
WHO: World Health Organization
