# Supplementary material for: Policy-makers’ views on translating burden of disease estimates in health policies: bridging the gap through data visualization
Source: Arch Public Health. 2021 Feb 4;79:17. doi: 10.1186/s13690-021-00537-z (PMC7863500; doi:10.1186/s13690-021-00537-z)
Supplement: Supplementary file 1 — Additional file 1. [file 13690_2021_537_MOESM1_ESM.docx]

# Appendices

**Appendix A. Interview Guide**

Hello ____,

My name is Amelia Lundkvist, and I am a master’s student in Health Economics, Policy and Management, at the Department of Public Health Sciences, at the Karolinska Institutet in Stockholm, Sweden.

As part of my Master’s program, I am conducting a thesis project entitled “**Examination of policy officers’ utilization of data visualization platform as part of their knowledge translation process: a cross-sectional qualitative study”.**

I would like to thank you in advance for your time and for your participation. Your insights as a technical policy officer are extremely valuable to us. Our interview should take about an hour, and we will touch on various topics regarding knowledge translation, data visualization, IHME and its GBD Compare tool.

If you have any questions during the interview, please do not hesitate to ask. If at any point you do not wish to answer a particular question, please just state your preference and we will move on. You can stop the interview or decide to no longer participate at any stage during the study.

We would like to record this interview, with your permission, and the content of the conversation will be transcribed. Any personal information will not be shared outside of this interview. Your statements will be kept confidential at all times and no names will be included in the transcribed text.

Do you have any questions?

[Pause here for 5-10 seconds to give the interviewee time to digest the information and reflect]

If you do not have any questions at this time, will you consent for me to start the interview and begin recording?

[Wait for interviewee reply]

[If the interviewee replies with yes, then to thank them for their agreement and announce that the interview will start]

1. To begin, can you tell me a bit about your professional background and work?
   1. Can you describe your level of experience and where you are currently in your career?
   2. How many years have you worked in health policy?
   3. [for researchers] Regarding your research, what is your research population?
      1. What is your research setting? Primary methodology?
2. We are interested in KT and data visualization. Before we go any further, what do you know about KT? How would you define it?
   1. Can you explain if KT is relevant to your work?
   2. If so, can you describe the level of relevance?
3. Where and how did you learn about KT?
   1. How would you describe the experiences of using KT in the process of decision-making?
   2. Can you give an example of a situation when you have used evidence to influence decision-making?
4. Do you see any factors impacting your desire or ability to engage in KT?
   1. Have you faced any difficulties obtaining or using evidence?
   2. In your experience, what are the major barriers to using evidence for decision-making?
   3. Are there any improvements you could recommend in order to make KT more useful for you in your work?
5. Is there anything else related to your involvement in KT that you think is important for me to know?
6. How would you define data visualization? What do you know about data visualization?
7. Regarding data visualization and its tools, have you used them before?
   1. Are there any tools you use regularly?
      1. If so, how frequently? Daily? Weekly? Monthly?
   2. How would you describe your experience using them?
8. What has your involvement been with IHME and the Global Burden of Disease study?
   1. How did you hear about it or get introduced to it?
9. Have you used IHME’s GBD Compare tool?
   1. Do you use it in your work?
      1. If so, for what purpose?
      2. How often do you use it?
10. What would you say are the strengths or advantages of GBD Compare and its visualizations?
11. Have you experienced any problems when using it?

1. Is there anything else regarding your involvement with data visualization that I should know?
2. Anything else about IHME and/or GBD Compare that you would like to share?
3. Finally, can you think of any colleagues that would be suitable and/or interested in participating in this study? If so, would you be willing to connect us? Thank you.
